# Research highlights for issue 5: disease spillover among natural and managed populations

**DOI:** 10.1111/eva.12271

**Published:** 2015-05-12

**Affiliations:** Evolutionary Applications

The recent epidemic of the Ebola virus is a particularly horrific example of the consequences of disease transmission between species. Spillover infection from populations of one species, in which a pathogen may be endemic, coevolved, and often less harmful, into populations of a novel host species has the potential to lead to epidemics of particularly virulent pests and pathogens. Understanding the probability of such spillover and the evolutionary, as well as coevolutionary, processes that occur after a cross-species transmission event occurs is key to predicting disease emergence and spread. This is especially important in the case of transmission between natural populations and managed ones, where disease emergence may have significant societal impact.

A classic example is the spillover of canine distemper virus from domestic dog populations into wild lion populations in the Serengeti, which has lead to a series of disease outbreaks and subsequent population declines. Using data collected across three decades, Viana et al. (2015) recently compared the disease dynamics of dog and lion populations to determine whether and how the two were linked. Their model suggests that although spillover from dog populations was the likely driver of disease in lion populations initially, the peak infection periods for each of the two species became increasingly asynchronous over time, suggesting a role for other reservoir species and/or evolution of the circulating viral strains. This work is an elegant example of the value of long term data sets, especially with the development and application of new statistical and modeling techniques, for examining the changing disease dynamics over time.

One powerful tool for uncovering patterns of spillover is the use of social and contact networks to study transmission, as recently reviewed by Craft (2015). Understanding transmission likelihood is a key first step in determining the selection acting on pathogen populations and the potential for host shifts and the piece outlines current methods for using information about contact within populations, for example as resulting from movement, sociality, or behavior, to help inform questions of disease transmission across livestock and interacting wildlife. Craft distinguishes the utility of social network analysis, in which contact structure within and/or among populations is described, and network modeling, a tool with which to simulate disease spread across a contact network, for predicting the risk and consequences of disease spread. She also discusses how human intervention of spatial structure and group size can alter the likelihood of transmission, both within and among populations, and therefore how spillover involving managed populations may differ from that among wild populations.

Another topical example of spillover from managed into natural populations is the case of wild pollinator exposure to viruses from commercial pollinators. A recent review by Manley et al. (2015) demonstrates the potential threat for movement of RNA viruses into wild pollinators from managed honeybee populations. As many of these viruses are known to be rapidly evolving, such spillover events can lead to pathogen adaptation to novel hosts and eventual host shifts. By collating evidence for viral spillover events among populations, the authors demonstrate the potential importance of cross-species transmission in shaping disease emergence, especially when there are shared ranges, niches or behaviors between managed and wild species. Work by McMahon et al. (2015) used data from a large-scale survey of co-occurring managed honeybee and wild bumblebee populations to explore correlations in prevalence and viral loads between the two, as might be expected if cross-species transmission was common. Although they found a significant association between prevalence of viruses in honeybees and bumblebees, they also report large species-specific differences in prevalence and load across the viruses examined, suggesting more data is needed to determine the direction of transmission.

Of course, not all spillover will have negative consequences; the introduction of natural enemies of pests from wild populations into managed ones can play a key role in keeping infestation levels down. For example, in the case of crops growing near forests, González et al. (2015) recently demonstrated that the diversity of natural enemies capable of controlling herbivores on soybean is dependent on the surrounding forest. By studying crop lands within the Argentine Chaco Serrano forest, they found that both the amount of forest cover and proximity to the forest were important indicators of the richness and taxonomic composition of natural enemy assemblages, including predators and parasitoid species. This highlights the potential benefits of connectedness between natural and managed populations for hindering enemy escape by emerging pests and emphasizes the difficulties of managing pest and pathogen spread between the two given the complex coevolutionary dynamics of communities.
